# The Value of Computed Tomography-Based Planning in Shoulder Arthroplasty Compared to Intra-/Interobserver Reliability of X-ray Planning

**DOI:** 10.3390/jcm13072022

**Published:** 2024-03-30

**Authors:** Martin Bischofreiter, Edanur Sacan, Michael Gattringer, Michael S. Gruber, Franziska L. Breulmann, Harald Kindermann, Philipp Heuberer, Georg Mattiassich, Reinhold Ortmaier

**Affiliations:** 1 Department of Orthopedic Surgery, Ordensklinikum Barmherzige Schwestern Linz, Vinzenzgruppe Center of Orthopedic Excellence, Teaching Hospital of the Paracelsus Medical University Salzburg, 5020 Salzburg, Austria; 2 Department of Orthopedic and Trauma Surgery, Clinic Diakonissen Schladming, 8970 Schladming, Austria; 3 Department of Orthopedic Sports Medicine, Klinikum Rechts der Isar, Technical University of Munich, 81675 Munich, Germany; 4 Department of Marketing and Electronic Business, University of Applied Sciences Upper Austria, 4400 Steyr, Austria; 5 healthPi Medical Center, 1010 Vienna, Austria; philipp@heuberer.at

**Keywords:** intra-observer reliability, inter-observer reliability, shoulder arthroplasty, X-ray, computer tomography, planning

## Abstract

**Background**: Reversed total shoulder arthroplasty (RTSA) is an established surgery for many pathologies of the shoulder and the demand continues to rise with an aging population. Preoperative planning is mandatory to support the surgeon’s understanding of the patient’s individual anatomy and, therefore, is crucial for the patient’s outcome. **Methods**: In this observational study, we identified 30 patients who underwent RTSA with two- and three-dimensional preoperative planning. Each patient underwent new two-dimensional planning from a medical student and an orthopedic resident as well as through a mid-volume and high-volume shoulder surgeon, which was repeated after a minimum of 4 weeks. The intra- and interobserver reliability was then analyzed and compared to the 3D planning and the implanted prosthesis. The evaluated parameters were the size of the pegged glenoid baseplate, glenosphere, and humeral short stem. **Results**: The inter-rater reliability showed higher deviations in all four raters compared to the 3D planning of the base plate, glenosphere, and shaft. The intra-rater reliability showed a better correlation in more experienced raters, especially in the planning of the shaft. **Conclusions**: Our study shows that 3D planning is more accurate than traditional planning on plain X-rays, despite experienced shoulder surgeons showing better results in 2D planning than inexperienced ones.

## 1. Introduction

Reversed total shoulder arthroplasty (RTSA) is an established procedure in the management of shoulder pathologies, and indications have grown from cuff-tear arthropathy to complex fractures, avascular necrosis, and also revision surgery of anatomic total shoulder arthroplasty (ATSA) in the last decades [1,2]. This is also reflected in the number of implanted RTSA with an almost three-fold increase in the period from 2012 to 2017 in the United States. Similar trends have been observed in other countries too [1,3]. With an aging population and rising requests for RTSA, there is also an increased expectation of the postoperative outcome. Therefore, the importance of precise preoperative planning cannot be emphasized enough. Although RTSA has been shown to improve pain and functional outcomes in patients with cuff-tear arthropathy, the surgery remains technically challenging, and meticulous preoperative planning, especially with the correct glenoid component positioning, is mandatory to prevent problems. It has been shown that malpositioning of the glenoid component accounts for 30–50% of all complications [4,5]. Traditionally, preoperative planning is performed using calibrated plain radiographs of the shoulder, which is cost- and time-effective and poses hardly any risks for the patient due to the low radiation dose. The disadvantage of 2D planning with plain radiographs is the lack of assessability of essential factors for the outcome of RTSA, like glenoid retroversion and bone stock, as well as the inclination of the glenoid and the scapular angle. As technology advanced, 3D preoperative planning using computed tomography (CT) scans and 3D software-assisted planning programs have been developed, offering a more precise understanding of the patient’s individual anatomy and consequently enabling the surgeon to perform more accurate planning [6]. This has been shown to be crucial for the accuracy of glenoid component positioning in terms of inclination and version of the glenoid baseplate, leading to better stability [4]. There is a general agreement among shoulder surgeons that superior tilt should be avoided in glenoid positioning because of higher rates of mechanical complications, including aseptic loosening of the glenoid baseplate, which was shown in several biomechanical studies [7]. However, there is still no consensus on the optimal tilt positioning of the glenoid base plate. Several studies have been conducted on whether the base plate should be placed neutral or with an inferior tilt on the effect of scapular notching, with contrary results [7,8]. Scapular notching, which is the occurrence of osteolysis on the inferior glenoid neck due to repetitive impingement of the humeral component can therefore only be prevented with inferior translation of the glenoid baseplate, lateralization of the glenosphere, and implantation of a larger glenosphere diameter [9]. Regarding the glenoid component version, a retroversion of no more than 10° should be aimed to gain maximal anterior stability. In contrast, more retroversion leads to reduced intrinsic stability. Intrinsic stability, the resistance to dislocation, can mainly be increased with a neutral version or slight anteversion of the humeral component. At the same time, too much anteversion should be avoided as it will limit external rotation. The glenoid version plays a subordinate role in terms of intrinsic stability [10]. Malpositioning of the glenoid can further be prevented with patient-specific instrumentation (PSI) guides, which have been shown to improve the radiological outcomes compared to standard instrumentation with 3D preoperative planning [11]. However, PSI guides, which are individually 3D-printed templates for the central K-wire application of the glenoid, did not impact clinical outcomes compared to 3D planning alone [12,13]. Furthermore, the implementation of new techniques like augmented reality can help to access these three-dimensional plans intraoperatively at any time using virtual reality goggles or displays and, therefore, increase intraoperative accuracy. With the advancement in 3D planning, there also comes the downside of high costs and high doses of radiation for CT scans. Although numerous studies have shown the efficacy of 3D preoperative planning, we hypothesize that conventional 2D planning remains a viable option for an experienced high-volume shoulder surgeon. Therefore, this study aimed to assess the inter- and intra-rater variability in the two-dimensional planning of RTSA, assessing the size of the glenoid base plate, glenosphere, and humeral stem, which were compared to the three-dimensional preoperative planning and the implanted RTSA components.

## 2. Materials and Methods

In this observational study, thirty patients who underwent RTSA were identified from the database at our institution (Ordensklinikum Linz-Barmherzige Schwestern, Austria). Approval from the responsible ethics committee was obtained before this study (Johannes Keppler University—Faculty of Medicine; 1263/2023).

The cases of patients matching the inclusion criteria, which were (1) surgery within the last two years and (2) implanted model Medacta^®^ reversed shoulder system, were selected randomly. Patients were excluded when an intraoperative model change or an additional intervention was performed. All patients routinely had a preoperative X-ray in two planes (ap/axial) with two-dimensional planning and a CT scan with three-dimensional planning. The CT scan was carried out according to a specific protocol required by Medacta^®^. The patient was in a supine position, with the arm in a neutral position and the elbow extended. The scan consisted of a spiral CT scan of the shoulder and the ipsilateral elbow with slices with a maximum of 1 mm. The data were then uploaded to the MyShoulder platform, and the planning was carried out together with a Medacta^®^ engineer.

Each of the thirty cases underwent new two-dimensional planning from four different raters: a medical student who had never planned or implanted any prosthesis before; an orthopedic resident who had never planned or implanted a reversed shoulder arthroplasty but had participated as an assistant during these surgeries; a mid-volume surgeon, with about 20–30 operations a year; and a high-volume shoulder arthroplasty surgeon, with over 50 operations a year (Table 1). The broad range of expertise within the group allowed for a thorough and well-rounded evaluation of CT-based planning in shoulder arthroplasty.

All the planners performed a two-dimensional planning of an RTSA with the Medicad^®^-System independently. The medical student and the orthopedic resident received a short technical introduction to the planning system and the shoulder model. Because only patients with a short stem shoulder arthroplasty were included, both of them were also instructed about the recommended bone canal filling ratio of 0.8 [14]. The three-dimensional planning was performed and authorized by the high-volume shoulder arthroplasty surgeon.

After a minimum of 4 weeks, all of the planners repeated the two-dimensional planning. This interval was chosen to reduce the bias by remembering the details of the already planned prostheses.

The evaluated parameters were the size of the pegged glenoid baseplate, glenosphere, and humeral short stem. The inter- and intra-rater reliability was then analyzed and compared to the 3D planning and the actual implanted prosthesis size.

## 3. Statistical Analysis

Light’s kappa for categorical data and the intraclass coefficient (ICC) for metric data were used to determine intraclass and interclass reliability. Cross-tabulations were also used to show each surgeon’s planning deviation between two time points. The chi-squared test was applied to the cross-tabulations for significance testing.

Deviations between planned and actual implants were determined in the same way.

A *p*-value less than 0.05 was considered significant and kappa or intraclass correlation coefficients (ICC) were calculated to determine the reliability of the correlation.

Kappa or ICC lies between 0 and 1, with 0 indicating no reliability among raters and 1 indicating perfect reliability.

According to Koo and Li, less than 0.50 indicates poor reliability, values between 0.5 and 0.75 indicate moderate reliability, values between 0.75 and 0.9 mean good reliability, and more than 0.9 indicates excellent reliability [15].

All analyses were performed using the R software package, version 4.3.0.

## 4. Results

### 4.1. Reliability of Baseplate Planning

While planning the baseplate, inter-rater reliability showed deviations ranging from 53.3% to 60.0%, as identified by the four different raters (Table 2). The findings remained uniform across all the assessors. This suggests a moderate level of inconsistency in the interpretation and planning of baseplate positioning. However, when using 3D planning, the deviations decreased to only 20% of cases, indicating a higher consistency among the raters. These findings demonstrate that CT-based planning in shoulder arthroplasty can improve inter-rater reliability compared to traditional X-ray planning methods.

Intra-rater consistency, on the other hand, exhibited a tendency towards improved reliability among more seasoned raters. Rater 1 showed a 56.7% and 70% deviation from planning to implanted parts at t1 and t2, respectively. In comparison, Rater 2 demonstrated deviations of 60.0% and 53.3%, rater 3 had deviations of 53.3% and 60.0%, and Rater 4 displayed deviations of 53.3% and 46.7%. Interestingly, the calculated kappa values indicated only moderate agreement for baseplate planning (κ = 0.5–0.7) in the least experienced rater group, whereas the evaluation by the three raters with greater experience suggested poor reliability (κ = <0.5; Table 3).

### 4.2. Reliability of Glenosphere Planning

The inter-rater reliability evaluation of glenosphere planning revealed significant differences in measurements between the raters, with deviations ranging from 33.3% to 83.3%. This suggests a lack of consensus among the raters regarding the optimal placement and positioning of the glenosphere. Interestingly, when utilizing 3D planning, the reliability of glenosphere planning improved significantly; deviations occurred in only 13.3% of cases, indicating a higher level of consistency.

Evaluation of intra-rater reliability revealed similar trends to those observed in baseplate planning. The difference between measurements at t1 and t2 and actual implanted parts were 83.3% and 80.0% in rater 1, respectively, 46.7% and 43.3% in rater 2, 33.3% and 50.0% in rater three, and finally, 40.0% and 26.7% in rater 4. These results indicate a lack of consistency in glenosphere planning among the raters, especially for those with less experience. The kappa values indicated poor reliability (Table 3).

### 4.3. Reliability of Stem Planning

Regarding stem planning, the raters’ inter-rater reliability was generally higher than the baseplate and glenosphere planning. Deviation percentages between planning and implantation ranged from 80.0% to 86.7%. It is worth noting that even though the inter-rater reliability was relatively higher for stem planning compared to the glenosphere and comparable to the base plate, there were still significant differences observed when compared to the final implant (very high share of deviations from planning to final size). Three-dimensional planning showed a noticeably worse result compared to the other components, with a deviation rate of 76.7%.

The evaluation of intra-rater reliability of stem planning revealed good or excellent reliability (κ > 75) in the planning of the shaft, with a significant correlation between planning in t1 and t2 (Table 3).

### 4.4. Intraclass Comparison with the Final Implanted Size

An intraclass comparison was conducted to assess the reliability of the raters’ planning in comparison to the final implanted size. Results showed significant differences in measurements between the raters and the final implanted size for all components (*p* < 0.05), with poor reliability of these measurements (Table 4). However, the correlation of the results of the 3D planning showed better agreement with the final implanted size compared to X-ray planning for the baseplate and glenosphere, indicating its potential value in improving the accuracy of shoulder arthroplasty planning.

The analysis of the final size of the implants revealed the superiority of 3D planning for the baseplate and glenosphere (Table 5).

### 4.5. Three-Dimensional planning Compared to Two-Dimensional X-ray Planning

Regarding baseplate planning, the 3D planning method exhibited higher consistency and reliability than traditional X-ray planning methods (deviations in only 20.0% of cases compared to 55.8%). Furthermore, the planning of the glenosphere also showed improved consistency with 3D planning, with deviations occurring only in 13.3% of cases compared to 50.8%. However, the analysis of the stem planning revealed that 3D planning did have a comparable rate of deviations compared to X-ray planning. In the study of the final size of the implants, 3D planning demonstrated superiority over X-ray planning for both the baseplate and glenosphere components (Table 5).

## 5. Discussion

In this study, we analyzed the intra- and interobserver reliability between four different planers with different skill levels in two-dimensional planning on preoperative x-rays (a.p. plane). The plannings were compared to a CT-based three-dimensional planning and the definitive implanted prosthesis.

We could show in our study that more experienced planners are more accurate regarding the definitive implanted prosthesis. However, the most accurate planning was the three-dimensional planning. All planners showed good intraobserver reliability, especially when planning the shaft. The medical student (planner with the least experience) showed the best intraobserver reliability over all prosthesis components. This could be explained by the fact that the student did not think about all the possible prosthesis configurations.

However, nowadays, two-dimensional planning via X-rays has become more dispensable because of the better availability of CT scans with two- and three-dimensional planning options. Most studies report on the latter and the increasing importance of glenoid positioning [4,6,11,16,17,18].

Nevertheless, we could show that especially the preoperative planning of the shaft on an X-ray (a.p. plane) had high intraobserver reliability compared to the base plate and the glenosphere. It also had the highest correlation to the definitive implanted prosthesis, though it showed poor reliability with a mean ICC of 0.352 (range 0.272–0.428). This was even worse with the base plate with a mean Light’s kappa of 0.167 (range 0.113–0.249) and the glenosphere with a mean Light’s kappa of 0.209 (range 0.001–0.349).

The high intraobserver reliability of the shaft lets us assume that the preoperative planning of the base plate and the glenosphere are more sophisticated than the shaft. The more experienced the surgeon was, the more accurate the planning was about reality.

Parsons et al. investigated the inter- and intrasurgeon variability in the preoperative planning of anatomic total shoulder arthroplasty. They performed the preoperative planning in forty-nine cases based on computed tomography scans. The planning was conducted by nine fellowship-trained shoulder surgeons using the Exac-techGPS platform (Exactech Inc., Gainesville, FL, USA). In this study, they also conducted a second planning between 4 and 12 weeks later. They only investigated the version, inclination, implant type, and implant size on the glenoid side. The interclass correlation coefficients for intersurgeon variability were 0.360 (Light’s kappa) for the implant size. Our population had a slightly lower correlation of 0.198 (Light’s kappa) for the base plate and glenosphere size. Regarding the shaft size, we had good reliability with a higher interclass correlation of 0.764 (ICC). However, this correlation was only based on the X-ray planning. Similar to the findings of Parson et al., we also propose that significant inter-surgeon and intra-surgeon variability in implant selection during preoperative planning suggests diverse approaches to achieving a surgical plan. Because of the differing experience levels among our planners, our group has already shown that experience indeed matters; with more experience, the planners demonstrate reduced variance, particularly concerning the shaft [19].

Many studies report the importance of correct alignment of the glenoid components in reversed shoulder arthroplasty. This can prevent complications such as scapular notching, loosening, and dislocations [20,21,22,23]. Favre et al. state that the retroversion of the glenoid components of less than 10 degrees reduces the risk of dislocation [10]. Superior tilt should be avoided to minimize micromotion and shear force at the interface of the glenoid bone and the base plate [24]. Furthermore, other authors recommended a tilt of the baseplate between 0° or 10° to minimize scapular notching/impingement [8,25]. However, a recent study from 2021 showed that an inferior tilt of 10° leads to medialization and increases impingement on the scapular neck external rotation with the arm at the side and adduction [26].

Due to the reasons mentioned above, it is clear that the traditional preoperative use of X-rays is no longer as essential. To adequately represent the glenoid, a preoperative CT scan is indispensable to perform shoulder arthroplasty on an evidence-based level. Considering that the anterior stability can be improved by changing the humeral component retroversion, CT scans, including the elbow, are becoming more relevant in determining the natural humeral retroversion [10]. In a systematic review in 2019, the authors included six studies with 237 cases and compared two-dimensional planning to three-dimensional planning in total shoulder arthroplasty. In their review, they showed no significant difference in the variability in glenoid measurements between 3D and 2D CT planning, with a difference in the version of 5° and 1.7° in inclination. However, in the 2D planning group, the posterior bone loss was underestimated by 52% compared to the 3D planning group. They also demonstrated that regardless of which planning was conducted, the definitive implanted components were larger than the planned ones (2D: 39%, 3D: 43%). In our cohort, we found similar results. The definitive implanted base plate in the X-ray planning group was 48.33%, and the shaft was 47.50% larger than planned. However, the implanted glenosphere was 44.17% smaller than planned. The 3D planning group showed 80.00% the same and 20.00% smaller implanted baseplates. None of them were planned too large. The implanted glenosphere was planned in 6.67% of cases, too large and too small. The definitive shaft was planned in 33.33% of cases too small and in 43.33% of cases too large. Therefore, the planning of the baseplate and the glenosphere is more accurate with 3D planning. However, the planning of the shaft is similar in both groups. The authors suggest the main advantages of 3D vs. 2D CT planning were improved preoperative foresight and accuracy in glenoid implantation, even though the measured differences were minor and without significant clinical relevance [27].

Preoperative X-rays can only provide inaccurate ideas about the extent of wear, particularly on the glenoid. Significant damage to the glenoid is often present, especially in cases of severe shoulder joint osteoarthritis. Knowledge of misalignments or wear is necessary to develop a good surgical strategy before the operation. Walch type B2 (biconcave) and C (hypoplastic) are especially difficult to handle in glenoid configurations. Therefore, 2D or 3D CT images are highly recommended to measure glenoid bone loss. Furthermore, good preoperative planning helps to ensure that the appropriate surgical instruments and implants are available before the surgery starts. Various options exist for addressing misalignments or wear on the glenoid. Apart from eccentric reaming or the use of bone blocks from autografts or allografts, wedged glenoid baseplates are also becoming increasingly popular [28,29,30,31,32]. Primary baseplate stability is necessary for good results in reversed shoulder arthroplasty. Several studies recommend a minimum of 50% contact area of the baseplate and the native glenoid [28,33,34]. Werner et al. proposed a minimum of 10 mm depth of the central peg into the native glenoid for good primary stability [33]. In addition, the divergence of the screws can improve the baseplate stability more than screw length or thickness [35]. With the help of preoperative computed tomography, precise planning (2D/3D) of the position of the glenoid baseplate and screws is possible. Nowadays, many companies also automatically calculate the contact area of the baseplate on the glenoid. Suppose this contact area is below 50% and cannot be improved with eccentric reaming in severe inclinations or version cases. Alternative procedures, such as bone blocks or augmented baseplates, are necessary in those cases. In a recent systematic review, the study group of Lanham et al. compared bone grafting (*n* = 401) and augmented baseplates (*n* = 251) for glenoid bone loss. They showed that the overall complication (11.7% vs. 11.8%), revision rates (4.5% vs. 3.7%), the range of motion (ROM), and the patient-reported and functional outcome scores were similar in both groups. The infection rate was higher in the bone-grafting group (1.9% vs. 0.7%). The authors assumed that the bone-grafting technique required more time to manipulate and contour the bone graft than the augmented baseplate technique. Furthermore, the scapular notching was more common in the bone-grafting group (24.6% vs. 4.7%). One possible reason mentioned by the authors was a failed ingrowth of the bone graft with absorption. Secondary notching could mainly occur when the bone graft was used for lateralization. Moreover, the component-loosening rate was also higher in the bone-grafting group (3.6% vs. 1.6%). Lanham et al. named the progression of notching as one possible explanation for the baseplate loosening [36].

Intraoperative glenoid positioning can be complex due to various factors, including bone stock, soft tissue condition, and surgeon expertise. As a result, several companies have developed new technologies such as patient-specific instrumentation, computer-assisted instrumentation, and augmented reality applications. These innovations aim to help surgeons minimize deviations from the planned position and achieve greater accuracy during surgery. Augmented reality, in particular, can provide surgeons with the real-time information necessary to transform preoperative planning into precise clinical outcomes. Though the technologies are improving rapidly, this does not necessarily translate into improved clinical outcomes [13,17,37,38].

Numerous studies have examined the different implementation tools from planning to reality. Several distinctions arise in comparing navigation systems and patient-specific instruments, although both technologies aim to enhance the intraoperative positioning of shoulder arthroplasty components. Patient-specific instruments require pre-production, which can take several weeks and involve associated costs. In contrast, navigation systems like augmented reality are linked to rental or one-time expenses but significantly reduce preoperative planning time [38,39,40]. However, research by Elsheikh et al. indicates that patient-specific instruments do not prolong the waiting time for surgery or the duration of the surgical procedure compared to standard instrumentation [13]. Previous cadaver studies have demonstrated promising outcomes regarding wire and glenoid placement using augmented reality applications. The group of Kriechling et al. conducted a feasibility study using a 3D scapula model, revealing a mean deviation of 2.7° ± 1.3° (95% CI 1.9°; 3.6°) and 2.3 mm ± 1.1 mm (95% CI 1.5 mm; 3.1 mm) in ten guidewires from the intended trajectory and entry point [40]. Subsequently, the authors reported in a later cadaver study achieving a mean deviation from the trajectory of 3.8° ± 1.7° (95% CI 2.6°; 4.9°) and from the entry point of 3.5 mm ± 1.7 mm (95% CI 2.4 mm; 4.6 mm) after two years [41]. Nevertheless, there is a lack of in vivo studies for augmented reality applications. In 2018, Gregory et al. released a technical report featuring an 80-year-old woman, demonstrating the potential effectiveness of augmented reality in reversed shoulder arthroplasty [42].

Based on our experience, utilizing Patient-Specific Instrumentation (PSI) often demands greater soft tissue release than augmented reality, primarily because PSI guides tend to be larger. However, this aspect should be discussed in PSI studies. In a systematic review and meta-analysis evaluating PSI for glenoid positioning, the authors noted potential challenges in accurately identifying the appropriate landmarks for guide placement and maintaining alignment during reaming. These difficulties were attributed to the bending and displacement of the guide pin [17].

## 6. Conclusions

Our study demonstrates that two-dimensional planning using X-rays has very low predictive power. More-experienced planners show better results than less-experienced ones. The preoperative three-dimensional planning based on CT scans was more accurate than conventional X-ray planning in AP regarding the definitive implanted components. However, they showed similar results in the planning of the shaft. Preoperative CT-scan-based planning (2D or 3D) is highly recommended to perform shoulder arthroplasty on a high and evidence-based level. Especially glenoid bone loss or deformities can be evaluated better, and the positioning and planning of the glenoid components are more accurate. Modern surgical techniques like navigation and augmented reality applications try to implement preoperative planning into the operating room. The limitations of this study include the limited amount of patients included. However, to evaluate the intra- and interobserver reliability of X-ray planning, we think the sample size is enough to determine trends in this observational study. Furthermore, we did not evaluate the postoperative prosthesis position as well as the patient’s outcome and whether this parameter correlates with the preoperative planning. This study raises opportunities for future research on factors contributing to the intra- and intersurgeon variability in the planning of RTSA, like patient characteristics and surgeon experience.

## Figures and Tables

**Table 1:** Demographic data of the four human raters.

Rater	Status	Age	Experience in Years	Experience in Cases (TSA)	Cases (TSA) per Year
1	Student	24	0	0	0
2	Resident	31	5	0	0
3	Specialist	33	10	85	20
4	Professor	38	14	>200	50

**Table 2:** Inter-rater correlation between the four raters (1–4 = surgeons, 5 = 3D planning).

			Base plate			Glenosphere			Shaft		
			No Deviations	Deviations	Total	No Deviations	Deviations	Total	No Deviations	Deviations	Total
Surgeon	1	Amount	13	17	30	5	25	30	4	26	30
		%	43.30%	56.70%	100%	16.70%	83.30%	100%	13.30%	86.70%	100%
	2	Amount	12	18	30	16	14	30	6	24	30
		%	40.00%	60.00%	100%	53.30%	46.70%	100%	20.00%	80.00%	100%
	3	Amount	14	16	30	20	10	30	5	25	30
		%	46.70%	53.30%	100%	66.70%	33.30%	100%	16.70%	83.30%	100%
	4	Amount	14	16	30	18	12	30	5	25	30
		%	46.70%	53.30%	100%	60.00%	40.00%	100%	16.70%	83.30%	100%
	5	Amount	24	6	30	26	4	30	7	23	30
		%	80.00%	20.00%	100%	86.70%	13.30%	100%	23.30%	76.70%	100%
Total		Amount	77	73	150	85	65	150	27	123	150
		%	51.30%	48.70%	100%	56.70%	43.30%	100%	18.00%	82.00%	100%
			Pearson-Chi-Quadrat: 12.702, df = 4, *p* = 0.013			Pearson-Chi-Quadrat: 32.036, df = 4, *p* < 0.001			Pearson-Chi-Quadrat: 1.174, df = 4, *p* = 0.882		

**Table 3:** Intra-rater reliability of human raters.

**Surgeon 1**			**Surgeon 2**	
Intraclass correlation coefficients for base plate, glenosphere, and shaft			Intraclass correlation coefficients for base plate, glenosphere, and shaft	
Characteristic	Light’s Kappa *|ICC **		Characteristic	Light’s Kappa *|ICC **
Base plate	0.525 (*p* = 0.005) *		Base plate	0.468 (*p* < 0.001) *
Glenosphere	0.443 (*p* = 0.009) *		Glenosphere	0.324 (*p* = 0.003) *
Shaft	0.917 (*p* < 0.001) **		Shaft	0.893 (*p* < 0.001) **
**Surgeon 3**			**Surgeon 4**	
Intraclass correlation coefficients for base plate, glenosphere, and shaft			Intraclass correlation coefficients for base plate, glenosphere, and shaft	
Characteristic	Light’s Kappa *|ICC **		Characteristic	Light’s Kappa *|ICC **
Base plate	0.462 (*p* = 0.027) *		Base plate	0.447 (*p* < 0.001) *
Glenosphere	0.463 (*p* < 0.001) *		Glenosphere	0.289 (*p* = 0.073) *
Shaft	0.891 (*p* < 0.001) **		Shaft	0.797 (*p* < 0.001) **

* Light’s Kappa; ** Interclass correlation coefficient.

**Table 4:** Correlation coefficients comparing planning at t1 with implanted components.

**Surgeon 1**			**Surgeon 2**	
Intraclass correlation coefficients for base plate, glenosphere, and shaft			Intraclass correlation coefficients for base plate, glenosphere, and shaft	
Characteristic	Light’s Kappa *|ICC **		Characteristic	Light’s Kappa *|ICC **
Base plate	0.149 (*p* = 0.291) *		Base plate	0.113 (*p* = 0.369) *
Glenosphere	0.001 (*p* = 0.987 )*		Glenosphere	0.267 (*p* = 0.053) *
Shaft	0.272 (*p* = 0.055) **		Shaft	0.412 (*p* = 0.012) **
**Surgeon 3**			**Surgeon 4**	
Intraclass correlation coefficients for base plate, glenosphere, and shaft			Intraclass correlation coefficients for base plate, glenosphere, and shaft	
Characteristic	Light’s Kappa *|ICC **		Characteristic	Light’s Kappa *|ICC **
Base plate	0.158 (*p* = 0.256) *		Base plate	0.249 (*p* = 0.033) *
Glenosphere	0.349 (*p* = 0.050) *		Glenosphere	0.221 (*p* = 0.214) *
Shaft	0.296 (*p* = 0.046) **		Shaft	0.428 (*p* = 0.008) **
**Surgeon 5 (Computer)**				
Intraclass correlation coefficients for base plate, glenosphere, and shaft				
Characteristic	Light’s Kappa *|ICC **			
Base plate	0.688 (*p* < 0.001) *			
Glenosphere	0.695 (*p* < 0.001) *			
Shaft	0.484 (*p* = 0.003) **			

* Light’s Kappa; ** Interclass correlation coefficient.

**Table 5:** Evaluation of planned size compared to implanted size.

Implant Greater than Planned (%)			
	Base Plate	Glenosphere	Shaft
Rater 1–4	48.33%	6.67%	47.50%
3D Planning	0.00%	6.67%	43.33%
Implant less than planned (%)			
	Base plate	Glenosphere	Shaft
Rater 1–4	3.33%	44.17%	35.83%
3D Planning	20.00%	6.67%	33.33%

## Data Availability

The data presented in this study are available on request from the corresponding author. The data are not publicly available due to ethical restrictions.
